# Comparative evaluation between conventional and digital orthodontic model analysis

**DOI:** 10.6026/973206300214399

**Published:** 2025-12-15

**Authors:** Rana Sarchanar Jamal, Parthiban Krishnaraj, Syed Mohammad Osama Ahsan, Lateef Shah Ali Syed, Abdulhakim Mahyoub Naser Alsharabi, Awaiz Ahmed, Md Kafeel Ahmed

**Affiliations:** 1 Dental Intern, College of Dentistry, Ajman University, United Arab Emirates; 2 Specialist Orthodontist, Gulf Asian Medical centre, Jubail, Saudi Arabia; 3 Department of Orthodontics and Dentofacial Orthopedics, Career Post Graduate Institutes of Dental Sciences and Hospital, Lucknow, Uttar Pradesh, India; 4 Registrar Orthodontist, Al Mashafi Medical Complex, Al Ahsa, Saudi Arabia; 5 Consultant Orthodontist, Al Mashafi Medical complex, Al Ahsa, Saudi Arabia; 6 Department of Pediatric Dentistry, Consultant, Dr. Awaiz's Dental Planet, Bidar, Karnataka, India; 7 Department of Periodontology and Implantology, MNR Dental College and Hospital, Sangareddy, Hyderabad, Telangana, India

**Keywords:** Digital orthodontic models, conventional plaster models, model analysis, intraoral scanning, diagnostic accuracy, orthodontic diagnosis

## Abstract

There is a need to validate whether digital orthodontic model analysis, using intraoral scans, provides equivalent accuracy, reproducibility, and time efficiency compared to conventional plaster model analysis. Hence, this cross-sectional study was conducted with 60 patients treated by orthodontists. The plaster models were taken through the acquisition of Impression of alginates and pouring and the intraoral scans were taken in a digital scanner. By two independent examiners, both models were examined on tooth measurements, arch measurements and the analysis by Bolton. Statistical evaluation was carried out using intraclass correlation coefficients, paired t-tests and Bland-Altman analysis. Digital orthodontic model analysis is as accurate as the traditional one, with better time efficiency and high reproducibility and as such, can be used in the clinic as an alternative to traditional plaster models.

## Background:

The analysis of orthodontic models is one of the foundations of diagnostic procedures and treatment planning in the modern orthodontic practice. Historically, the plaster dental casts produced by either alginate or elastomeric impressions have been the gold standard of extensive assessment of malocclusion, tooth size discrepancies and spatial relationships of the dental arches [1]. These physical models offer physical real-life models that can be handled, measured and kept by clinicians as permanent data of the pre-treatment, the intermediate and the post-treatment dental morphology [2]. Nevertheless, traditional plaster models have the following limitations such as the requirement to store them, the vulnerability to physical damage, challenges in digital communication and the labor-consuming manufacturing procedures [3]. The emergence of the digital technology has significantly changed the way dentists and their respective fields of discipline approach diagnosis. Three-dimensional imaging systems and intraoral scanners have made it possible to obtain the most precise digital impressions, which allow making a virtual dental model [4]. There are many benefits of digital orthodontic models such as effective e-storage, ease of communication, integration with computer-aided design and manufacturing systems and high treatment simulation [5]. Additionally, the use of digital workflows eliminates material costs that are concerned with impression materials and stone models in addition to minimizing chair time and patient suffering [6]. Recently, there has been the research on the validity and dependability of the digital models versus the traditional models. Surveys by Grünheid *et al.* showed that digital models have a high reproducibility when linear measurements are involved with negligible differences when compared to the plaster models [7]. On the same note, a study by Wiranto *et al.* showed that there was high validity of digital models in orthodontics measurements implying their clinical applicability [8]. On the other hand, other researchers have found that there are slight differences in certain measurements especially in the posterior parts of the body where accessibility of the scanner can be problematic in access [9]. These differences and the effects they have on the decisions in the treatment planning are the issues of ongoing research [10]. Although the scientific literature on the benefits of digital orthodontics is increasing, there is a lack of comparative studies with multiple parameters of measurement, reliability studies and time-saving analyses. Also, it is limited by the fact that the generalizability of the current findings is limited due to the differences in scanner technologies, software platforms and methods [11]. Hence, a systematic comparison with standardized measurement protocols and strong statistical analyses is necessary. Therefore, it is of interest to conduct a comprehensive comparative evaluation of conventional plaster model analysis and digital orthodontic model analysis.

## Specific aims included:

[1] Assessing the accuracy of linear measurements obtained from modalities,

[2] Evaluating intra-examiner and inter-examiner reliability,

[3] Comparing time efficiency between the two methods

[4] Determining the clinical acceptability of digital models as alternatives to conventional plaster models in orthodontic diagnosis and treatment planning.

## Materials and Methods:

## Study design and setting:

The G 3.1.9.7 software (paired t -test) was used to determine the sample size based on an effect size of 0.5, alpha error of 0.05 and power of 0.85. A minimum of 60 participants was estimated to account for a 10 percent dropout.

## Inclusion and exclusion criteria:

## Inclusion criteria:

Patients aged between 15 and 30 years with full orthodontic treatment and complete first molar to first molar in arches, not receiving any previous orthodontic treatment and willing to take part in the study.

## Exclusion criteria:

Proximal restorations or crowns that influence the mesiodistal tooth width, extreme crowding that cannot allow accurate tooth identification, craniofacial syndromes or cleft lip and palate, absence of permanent teeth (except third molars), poor-quality impressions or scans, which need to be repeated.

## Sample preparation and model preparation:

## Conventional models:

The impressions of the maxillary and mandibular were taken with the help of the standard stainless-steel perforated trays with the subsequent manufacturer instructions of a product called Tropicalgin (Zhermack, Italy). The impressions were immediately disinfected with 2 percent glutaraldehyde solution and poured in less than 15 minutes using Type III dental stone (Kalabhai Karson Pvt. Ltd., India) based on the ratio between powder and water. The models were clipped according to standard procedures with special identification codes on them.

## Digital models:

The iTero Element 5D scanner (Align Technology, USA) was used to obtain intraoral digital scans of one calibrated operator. The scanning protocol was which consisted of buccal, occlusal, lingual surfaces of both arches and the registration of the bite on the buccal surface. The files in the form of Standard Tessellation Language (STL) were exported and stored in a safe database. The visualization and analysis of digital models were performed with the help of the Ortho Analyzer software (3Shape, Denmark).

## Measurement protocol:

All measurements of conventional and digital models were done by two independent calibrated orthodontists with at least five years of clinical practice experience. Examiners were subjected to calibration sessions at a level of intraclass correlation coefficient of above 0.90 before the study. After two weeks the measurements were again taken to determine intra-examiner reliability.

## The measured parameters included:

[1] Mesiodistal tooth width of all teeth (first molar to first molar) (24 teeth/patient).

[2] Maxillary and mandibular arch length (length between mesial point of the first molars to the mid-point on the central incisors)

[3] Maxillary and mandibular intercanine width (spacing between the tips of canines)

[4] Maxillary and Mandibular intermolar width (spacing between the mesiobuccal cusp tips of the first molars)

[5] The anterior and overall ratios of Bolton.

The digital caliper (Mitutoyo Corporation, Japan) with 0.01mm precision was used to find conventional measurements. The software had integrated measurement tools that were used to measure digital values at the same precision.

## Statistical analysis:

The SPSS Statistics version 26.0 (IBM Corp., USA) was used to analyze the data. Shapiro-Wilk test was used to test normalcy. Means and Standard deviations were used as descriptive statistics. There were paired t-tests comparing measurements of conventional and digital method. Intra-examiner and inter-examiner reliability was determined by intraclass correlation coefficients (ICC) with 95 percent intervals. Bland-Altman plot was used to evaluate method agreement. Independent t-test was used to compare efficiency in terms of time. The statistical significance was predetermined to be p<0.05.

## Results:

The final sample comprised 60 patients (26 males, 34 females) with mean age 21.4±4.2 years. All participants completed both conventional and digital model acquisitions without complications. No models were excluded due to quality issues. Intra-examiner reliability demonstrated excellent agreement for both conventional (ICC: 0.95-0.97) and digital measurements (ICC: 0.96-0.98). Inter-examiner reliability was similarly high, with ICC values of 0.93-0.96 for conventional and 0.94-0.97 for digital measurements, indicating minimal measurement variability between examiners. Table 1 (see PDF) presents mesiodistal tooth width measurements. No statistically significant differences were observed between conventional and digital methods for any tooth (p>0.05), with mean differences ranging from 0.02mm to 0.14mm. Table 2 (see PDF) demonstrates arch dimension comparisons. Both arch length and width measurements showed no significant differences between methods (p>0.05), with excellent correlation coefficients. Table 3 (see PDF) presents Bolton's ratio analysis and time efficiency comparison. Bolton's ratios showed no significant differences, while digital analysis demonstrated significantly reduced analysis time. Bland-Altman plots revealed excellent agreement between conventional and digital methods. For mesiodistal tooth width measurements, 95% of differences fell within ± 0.3mm limits of agreement. Arch dimension measurements demonstrated limits of agreement within ± 0.4mm, indicating clinically acceptable concordance.

## Discussion:

In the current study a detailed comparative analysis of traditional plaster models and digital orthodontics models was done in terms of various measures of the model such as measurement parameters, reliability measures and time metrics. The results indicate that digital analysis of orthodontic models is as accurate as the traditional ones and has considerable benefits in the regard of time saved and optimization of the working process. The lack of statistically significant differences in the mesiodistal tooth width measurements of conventional and digital models supports the results of other studies carried out in the past. Such findings are consistent with systematic reviews of Mangano *et al.* that determined that intraoral scanners are highly accurate dental calculations with error margins usually lower than clinically significant levels [12]. The average difference between the two groups in our research (0.02- 0.14mm) does not exceed the scope of clinical acceptability, with any variation less than 0.5mm being thought to be inconsequential in orthodontic treatment planning [13]. The correlation coefficients (r>0.97) are also excellent, which further support the exchangeability of the two methods to be used in everyday clinic routines. Arch measurements were taken, such as the arch length, intercanine width and intermolar width, which showed that the two modalities were in very good agreement. These results can be discussed in terms of findings of Rossini *et al.* who found out that digital models can produce an accurate representation of the arch morphology that can be used to conduct diagnostic tests [14]. Such a high level of accuracy of digital measurements is explained by the use of sophisticated scanning technologies that can measure the topography of the surface with a high level of precision, usually reaching values of trueness of less than 50 micrometers in the intraoral scanner [15]. Further, there are the removal of dimensional variations of material setting to an impression, stone expansion and manual trimming in computerized processes, which add to the increased consistency in measurements. The present findings are consistent with the results of Sharma *et al.* who demonstrated that digital study models provide tooth measurements that are as accurate, reliable, and reproducible as those obtained from conventional casts [16]. This finding has significant clinical implications because the ratios used by Bolton directly affect the treatment mechanics, the choices made in extraction and the interproximal reduction need. The power to conduct the correct analysis of Bolton digitally simplifies the process of diagnosing and allows connecting it with digital treatment plans and clear aligner fabrication systems. The fact that the analysis of the digital model is much more efficient in terms of time is a great practical benefit. We have shown that digital analysis took 46.7% less time than the traditional ones (p<0.001). This is reduction of the impression pouring, model trimming and physical manipulation time. Earlier research reports have also provided increased efficiency of workflow using digital systems [17]. This time savings is associated with clinical productivity, a decrease in the cost of laboratory services and the possibility of chairside treatment planning and communication with the patient based on the three-dimensional visualizations. The good intra-examiner and inter-examiner reliability of the two methods is a confirmation of the consistency of measurements. Significantly, there was a marginally higher level of ICC with reference to digital measurements, which this could be explained by the use of standardized measurement instruments embedded in analysis software that reduce the error of human factor and deviation in methods of measurements. Digital environment offers steady lighting, magnification and landmark identification features, as opposed to traditional measurements that can be subject to caliper placement and visual parallax. Practically, digital models also have other benefits on top of accuracy of measurements. The need to store plaster models physically occupies a lot of physical space, which is overcome by electronic storage in orthodontic practices, which has always been a major problem [18]. Digital models serve as the means of remote consultation, interdisciplinary cooperation and longitudinal monitoring of treatment based on superimposition analyses. More so, incorporation with three-dimensional printing technology allows on-demand physical model construction where it is needed to integrate the advantages of both paradigms. Nonetheless, there are some restrictions that should be mentioned. Some practices have a major financial limitation in the initial cost of investing in an intraoral scanning equipment and software. There are learning curves related to methods of scanning and use of software which require specialized training. Also, the scanning technology that we used was modern, although there are differences in the accuracy, speed and clinical management of various scanner systems. The following research needs to be conducted and compare several scanning platforms and test the data integrity and software compatibility over time.

## Conclusion:

Digital model analysis provides accuracy comparable to conventional plaster models, with added advantages of time efficiency and reliability. Its improved storage, communication and integration with modern digital treatment planning make it clinically practical and beneficial. With advances in scanning technologies, digital orthodontic models are likely to become the routine standard in contemporary orthodontic practice.
